# Brain and Cardiac Concomitant Localization of the Hydatid Cyst

**DOI:** 10.1155/2020/4829496

**Published:** 2020-08-18

**Authors:** Amal El Ouarradi, Sara Oualim, Ilham Bensahi, Meriem Elkouhen, Imane Abouloiafa, Mohamed Sabry

**Affiliations:** ^1^Department of Cardiology, Mohammed VI University of Health Sciences, Cheikh Khalifa Hospital, Casablanca, Morocco; ^2^Department of Cardiology, Regional Hospital of Benimellal, Beni Mellal, Morocco; ^3^Department of Radiology, Regional Hospital of Benimellal, Beni Mellal, Morocco

## Abstract

Hydatid cyst is a parasitic infestation that is usually observed in the liver and lungs. The localization in the brain and the heart is exceptional. Here, we report a 11-year-old boy who was diagnosed to have two large hydatid cysts of the heart and brain. We discuss this unusual presentation of hydatid cyst and its management.

## 1. Learning Objective

By reporting a rare case of brain and cardiac concomitant localization of the hydatid cyst, we underlie this atypical clinical presentation and treatment difficulties.

A hydatid cyst is a parasitic illness that is frequently seen in rural areas where husbandry is widely practiced. It can affect any organ and is usually observed in the liver (∼75%) and lungs (∼20%) [1]. The location in the brain and heart is exceptional. This case is particularly interesting because of the rarity of concomitant large hydatid cyst in two uncommon localizations: heart and brain.

## 2. Case Report

We received an eleven-year-old boy with Down's syndrome and mental retardation and psychiatric trouble (hyperexitability). His parents noticed dyspnea and decreased exercise tolerance. The family lives in a rural area in companion with domestic animals.

**Figure 1:**
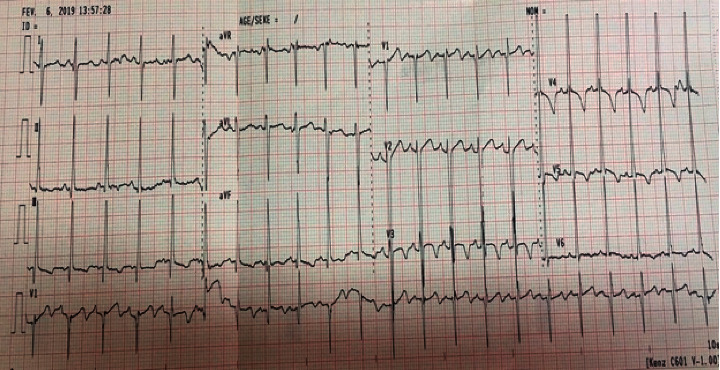
ECG: RRS with HLV and negative T wave.

**Figure 2:**
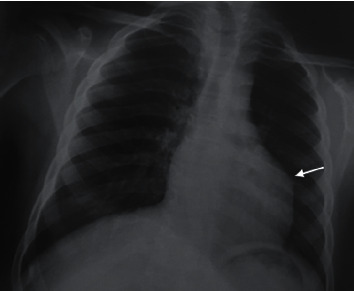
Frontal chest X-ray: globular appearance of the lower left arch (arrow).

**Figure 3:**
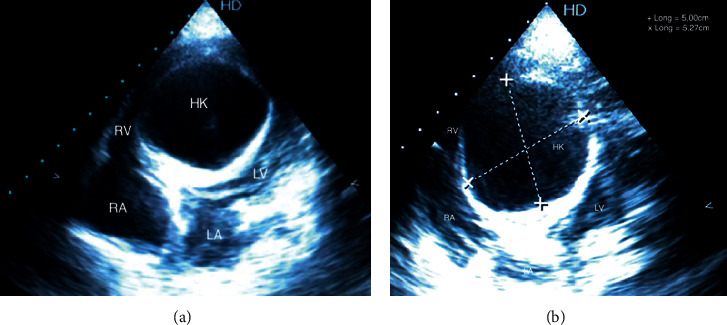
Echocardiography: transthoracic apical 4-chamber view showing a large cystic mass splitting the interventricular septum (50 mm/52 mm). HK: hydatid cyst, LV: left ventricle, LA: left atrium, RV: right ventricle, RA: right atrium.

The boy cannot express due to mental disorder. On physical examination, he was irritable. However, his parents reassured us that he was always like this with strangers. Cardiopulmonary auscultation was normal. Electrocardiogram showed sinus rhythm with 110 beats per minute and systolic ventricular hypertrophy (Sokolow score at 37 mm), with a negative T wave (Figure 1). Frontal chest X-ray showed no lung parenchymal abnormality, no pleural effusion, and normal cardiothoracic ratio with globular appearance of the lower left arch (Figure 2). Transthoracic echocardiography revealed a large cyst measuring 50 mm/52 mm in the middle part of the interventricular septum, crushing left and right ventricular (Figure 3). His left ventricular systolic function was altered with a low left ventricular ejection fraction (45%). Minimal tricuspid regurgitation was noted. There was no pericardial effusion.

**Figure 4:**
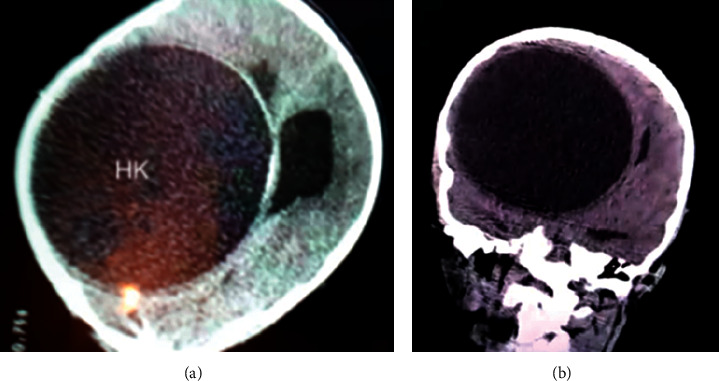
Brain scanner: large brain hydatid cyst in the right fronto-parieto-temporal lobe with important shifting of the cerebral hemisphere (10 cm/9.5 cm). HK: hydatid cyst.

On abdominal ultrasound, no cysts were observed in the liver, kidneys, or spleen. Hydatid serology was positive (hemagglutination greater than 1/5,000 (threshold 1/320), ELISA was positive, and optic density was 1.034 (threshold 0.275)). We started preoperative albendazole (at a posology of 15 mg/kg/day or 400 mg per day), and we decided to proceed with surgical removal. Two weeks after the diagnosis of cardiac hydatid cyst, the patient made a severe neurological impairment and was admitted to the emergency for symptoms of increased intracranial pressure, nausea, and vomiting. A brain-CT demonstrated a very large cerebral cyst with signs of mass effect (Figure 4). The cardiac cyst has not changed its appearance. He was operated on by a left fronto-temporo-parietal craniotomy. The cyst was removed by the Dowling–Orlando technique with the aid of gravity without rupture. Histopathological examination confirmed the diagnostic of hydatid cyst, and the medical therapy using albendazole was pursued.

We were planning to remove the cardiac hydatid cyst one month after the brain surgery. Unfortunately, the patient had a shock and died immediately after the first surgery.

## 3. Discussion

Hydatid cyst is a parasitic infestation caused by the larval stage of the different species of the tapeworm *Echinococcus genus*. The most common form worldwide including Morrocco is cystic echinococcosis that is chiefly caused by *Echinococcus granulosus*. It is endemic in Mediterranean countries, the Middle East, South America, and Australia [1].

In Morrocco, the incidence of cystic echinococcosis is 5.2 cases per 100,000 inhabitants with a predominance in females (sex ratio M/F = 0.66) and young adults (59.1% of hydatidosis have been diagnosed in patients aged 15 to 49 years) [2].

Like many other parasitic infestations, the course of *Echinococcus* is complex. The worm has a life cycle that requires definitive hosts and intermediate hosts. Definitive hosts are normally carnivores such as dogs and cats while intermediate hosts are usually herbivores such as sheep or cattle. *Echinococci* are transmitted to intermediate hosts via the ingestion of eggs as humans do, whereas they are transmitted to definitive hosts by means of eating infected, cyst-containing organs. Humans are accidental intermediate hosts that become infected by handling soil or dog excrement that contains eggs or by ingestion of food contaminated by the ova of the parasite [1].

The potential risk factors of cystic echinococcosis are the regional epidemic, the rural environment with hygiene practices, social conditions, education, and the young age. In fact, 29% of the cases were found in children younger than 14 years [1]. Our patient has all of these risk factors. However, the diagnosis was delayed because of the lack of expression by the patient (mental retardation and Down's syndrome) [3].

The liver and lungs are the organs most affected by parasitosis. Hepatic hydatidosis is by far the most common (75%), followed by lung locations (20%). Still, hydatidosis can be found in any site of the body: brain, muscle, heart, kidney, spleen, peritoneum, and so on [1]. The hydatid cyst of the central nervous system or cardiac system is unusual with a reported frequency of 1-2% for brain, and 0.5–2% for cardiac localization of all cases with hydatid cyst disease [1, 4]. The most common cardiac locations are the left ventricular wall (60%) followed by the right ventricle (10%), pericardium (7%), left atrium (6–8%), right atrium (4%), and the interventricular septum (4%). In 50% of such cardiac cases, there is multiple organ inclusion. Patients with a cardiac hydatid cyst may be asymptomatic, while other patients can develop symptoms because of the cyst's compression of a coronary artery or conduction system. Cardiac hydatid cysts may lead to serious complications including cyst rupture, anaphylactic shock, tamponade, pulmonary, cerebral or peripheral arterial embolism, acute coronary syndrome, dysrhythmias, infection, ventricular or valvular dysfunction, and sudden death. Electrocardiogram abnormalities such as Q waves and inverted T waves in the inferior leads may also occur as signs of ventricular hypertrophy. When present, these signs should raise the suspicion of cardiac hydatidosis in patients living in endemic regions even when clinical symptoms are absent. Echocardiography is a noninvasive procedure which provides important findings: size and number of cysts, cyst locations, and relationships with adjacent structures. Cardiac CT and cardiac MRI (magnetic resonance imaging) show the anatomic extent and position of the mass and its relationship with the cardiac chambers [5–7].

Brain localization is usually single, supratentorial, and intraparenchymal. The cysts do not produce serious symptoms until they grow to large dimensions. A cyst's clinical symptoms depend on its localization and the dimensions of the lesion in the central nervous system. Headaches and throwing up are the most frequently observed symptoms [8, 9].

Symptoms develop slowly, and neurological deficits generally appear in the late period as a result of an increase in intracranial pressure. Papillary edema is frequently seen in children, while focal symptoms, such as hemiparesis, paraphasia, hemianopsia, and seizures, are frequently observed in mature individuals and depend on the localization of the cyst [8, 9].

Psychiatric disorders (behavioural disorders, aggression, and delirium) have also been reported in the literature. In our patient, perhaps, his irritability and agitation are related to his cerebral hydatid cyst, but this was difficult to prove because of his mental retardation. He later presented with signs of intracranial hypertension [10].

A clinical diagnosis is made according to epidemiological findings, the patient's history and clinical symptoms, any morphological lesions that are discovered by ultrasonography (US), CT, and MRI, and serological tests [2, 4].

The tests for diagnosis of hydatidosis include detection of IgE and IgG by western blot and molecular analysis of the excised tissues. Sequencing of the DNA polymerase chain reaction of contents from the cyst could also be performed as more specific test [2, 4].

Management of disseminated echinococcal disease is complex, which requires a multidisciplinary approach. Hydatid disease should be treated with either albendazole and surgery or albendazole and PAIR (puncture, aspiration, injection of scolicidal agent, and reaspiration) to extract the cyst without any complications [11, 12].

As of high frequency of recurrent hydatid disease after treatment with only surgery or only albendazole, multidisciplinary approach is recommended, and continuing medical treatment for at least 3 months postoperatively is also suggested [10]. In patients with multiorgan hydatid cysts, surgical removal of all cysts at the same time is recommended if possible. Treatment with mebendazole or albendazole also has very effective results in patients with multiple or inoperable cysts [13, 14].

In our case, we started with the medical treatment, and then we performed the cerebral hydatid cyst surgery given the urgency as the patient presented with signs of cerebral engagement. An ablation of the cardiac hydatid cyst was planned in a second time. Unfortunately, the postoperative shock state probably due to anaphylactic shock took the patient away.

In the literature, causes of rapid clinical decline involve a wide range of mechanisms including anaphylaxis (with or without cyst rupture), cardiac outflow obstruction or conduction tract disturbance, pulmonary and cerebral embolism, pericarditis, cardiac tamponade, myocardial ischemia, pulmonary hypertension, peritonitis, hollow organ perforation, intracerebral mass effect, obstructive hydrocephalus, seizures, and cerebral ischemia/infarction. The autopsy helps determine the exact cause of death. It was not performed on our patient because the family refused [15, 16].

## 4. Conclusion

When a hydatid cyst is found, whatever the localization is, it is necessary to assess the extension by a brain-CT, chest X-ray, echocardiography, and abdominal echography. Atypical localization can be found and can change the therapeutic vision, especially in children who live in endemic regions. Medical treatment must be started without delay along with surgical removal of all cysts if possible.
